# Embelin as Lead Compound for New Neuroserpin Polymerization Inhibitors

**DOI:** 10.3390/life10070111

**Published:** 2020-07-11

**Authors:** Cristina Visentin, Loana Musso, Luca Broggini, Francesca Bonato, Rosaria Russo, Claudia Moriconi, Martino Bolognesi, Elena Miranda, Sabrina Dallavalle, Daniele Passarella, Stefano Ricagno

**Affiliations:** 1 Dipartimento di Bioscienze, Università degli Studi di Milano, Via Celoria, 26, 20133 Milan, Italy; cristina.visentin@unimi.it (C.V.); luca.broggini@unimi.it (L.B.); martino.bolognesi@unimi.it (M.B.); 2 Dipartimento di Scienze per gli Alimenti, la Nutrizione e l’Ambiente, Università degli Studi di Milano, Via Celoria, 2, 20133 Milan, Italy; loana.musso@unimi.it (L.M.); sabrina.dallavalle@unimi.it (S.D.); 3 Dipartimento di Chimica, Università degli Studi di Milano, Via Golgi, 19, 20133 Milan, Italy; francesca.bonato@unimi.it (F.B.); daniele.passarella@unimi.it (D.P.); 4 Dipartimento di Fisiopatologia Medico-Chirurgica e dei Trapianti, Università degli Studi di Milano, Via Fratelli Cervi, 93, 20090 Segrate, Italy; rosaria.russo@unimi.it; 5 Dipartimento di Biologia e Biotecnologie ‘Charles Darwin’, Sapienza Università di Roma, Piazzale Aldo Moro, 5, 00185 Rome, Italy; moriconi.cla@gmail.com (C.M.); mariaelena.mirandabanos@uniroma1.it (E.M.); 6 Istituto Pasteur—Cenci Bolognetti Foundation, Sapienza Università di Roma, Piazzale Aldo Moro, 5, 00185 Rome, Italy

**Keywords:** protein polymerization, drug design, neurodegeneration

## Abstract

Familial encephalopathy with neuroserpin inclusion bodies (FENIB) is a severe and lethal neurodegenerative disease. Upon specific point mutations in the *SERPINI1*gene-coding for the human protein neuroserpin (NS) the resulting pathologic NS variants polymerize and accumulate within the endoplasmic reticulum of neurons in the central nervous system. To date, embelin (EMB) is the only known inhibitor of NS polymerization in vitro. This molecule is capable of preventing NS polymerization and dissolving preformed polymers. Here, we show that lowering EMB concentration results in increasing size of NS oligomers in vitro. Moreover, we observe that in cells expressing NS, the polymerization of G392E NS is reduced, but this effect is mediated by an increased proteasomal degradation rather than polymerization impairment. For these reasons we designed a systematic chemical evolution of the EMB scaffold aimed to improve its anti-polymerization properties. The effect of EMB analogs against NS polymerization was assessed in vitro. None of the EMB analogs displayed an anti-polymerization activity better than the one reported for EMB, indicating that the EMB–NS interaction surface is very specific and highly optimized. Thus, our results indicate that EMB is, to date, still the best candidate for developing a treatment against NS polymerization.

## 1. Introduction

The serpinopathies are a group of conformational diseases characterized by the accumulation of a misfolded member of the serpin (serine protease inhibitor) superfamily into large inclusion bodies that cause cellular toxicity [1]. Familial encephalopathy with neuroserpin inclusion bodies (FENIB) is a member of this group of pathologies. FENIB is a rare and autosomal dominant genetic disease with fatal outcome [2], which presents as consequence of point mutations in the *SERPINI1*gene coding for neuroserpin (NS) [3,4,5,6]. Mutant NS accumulates within the endoplasmic reticulum of neurons causing progressive neurodegeneration, with consequent epilepsy, cognitive impairment and dementia as clinical symptoms [2,3,4,5,6]. The amount of inclusions and the severity of the pathology, as well as the date of onset, depend on the specific mutation present in the patient [4,7]. To date, there are six reported mutations responsible for FENIB: S49P, L47P, S52R, H338R, G392E and G392R [2,4,5,6].

NS is mainly expressed in the central nervous system [8], where is involved in several cellular and developmental processes including synaptic and axonal plasticity, learning and memory [9,10,11]. As a serpin, NS mainly inhibits tissue plasminogen activator (tPA) [12]. Substrate recognition and inhibition are mediated by a long disordered loop, named reactive center loop (RCL), that is specifically recognized and cleaved by tPA. Upon cleavage, there is a transition from disorder to order in the N-terminal part of the RCL, which is inserted in beta-sheet A of NS and drags the tPA molecule to the opposite side of the NS molecule, before the hydrolysis of the acyl NS-tPA complex. The geometry of the protease’s active site is distorted in this process preventing the completion of the proteolytic reaction [13]. Typically, such acyl–enzyme, serpin–protease, complexes are not hydrolysable thus very stable over time, on the contrary the NS-tPA complex is short-lived and is hydrolyzed within minutes [14,15]. After cleavage, the RCL, which is usually exposed in the native (Nat) conformation, gets inserted into the beta sheet A between strands 3A and 5A. This conformation is referred to as cleaved (Cl) NS and is inactive. NS shares the typical serpin fold with the other members of the superfamily. The exposure of the RCL loop makes the Nat fold metastable and this accounts for its intrinsic propensity to adopt more stable conformations such as the latent (Lat) and the polymeric forms (Pol). Lat is extremely stable, but inactive: in this monomeric conformation the RCL is uncleaved, but nevertheless inserted in the beta sheet A, as observed for Cl [16].

NS, as many secreted proteins, is glycosylated, presenting two N-glycosylation chains in positions N157 and N321 that decrease spontaneous polymerization in vitro and in vivo [17,18]. Indeed, alteration of the glycosylation pattern due to their removal or pathologic mutation leads to enhanced polymers (Pol) formation [17,18]. The molecular structure of serpin Pol is still under debate and several models have been proposed, mainly for alfa-1 antitrypsin and alfa-1 antithrombin, two prototypical serpins for molecular studies. In all such models, the transition of the RCL from the disordered exposed conformation to the highly structured beta conformation is a paramount step. Specifically, the RCL inserts in beta sheet A of neighboring monomers as part of the intermolecular link [19,20] or intramolecularly allowing a larger domain swap of the C-terminal stretch of the protein [21]. This latter model is gaining momentum. Like the ones formed by other serpins, NS polymers are long, flexible and unbranched [22], and spectroscopic data indicate that the Nat to Pol transition implies a gain in secondary structure content [23,24].

FENIB, as other serpinopathies, is an incurable disease and there are no active clinical trials for FENIB therapies. Several approaches have been proposed for the treatment of alfa-1 antitrypsin deficiency, the most common serpinopathy [16], but no cure for this conditions has been reported yet. Chemical chaperones, peptides related to the RCL and small molecules have been proposed, however a specific and efficient polymerization inhibitor has not been identified so far [16]. We recently reported the ability of embelin (EMB) to prevent NS polymerization and to dissolve preformed NS polymers [25]. In vitro, EMB binds to all NS conformations destabilizing both the Nat and Lat forms and resulting in the accumulation of small and soluble oligomers (Olig) [25]. EMB is the major active constituent of fruits from *Embelia ribes* and is chemically known as 2,5-dihydroxy-3-undecyl-1,4-benzoquinone. This molecule was long known in Asian traditional medicine [26], and its pharmaceutical potential against central nervous system pathologies has been extensively investigated in the literature [27]. It is particularly interesting for its capability to cross the blood brain barrier and to elicit its effect directly in the central nervous system [28].

Here we show that in presence of EMB concentrations lower than 0.75 mM NS forms increasingly bigger aggregates; however, at 0.12 mM—the lowest concentration tested—a residual effect is still detectable. Moreover we observed a reduction in NS polymeric accumulation in COS-7 cells treated with EMB, however our results suggest that this reduction was not related to the expected anti-aggregation effect of the EMB molecule but was mainly due to the activation of the proteasome system. We thus we performed a systematic chemical modification of the EMB scaffold to identify EMB analogs with better anti-polymerization properties and tested the effects of each EMB analog against NS polymerization in vitro. Despite its simple structure, the chemical properties of EMB seem to be already optimal for NS polymerization inhibition, and no better molecule was identified. Thus, EMB remains the best starting point for the future development of a treatment against FENIB.

## 2. Materials and Methods

### 2.1. NS Expression and Purification

*E. coli* BL21 Rosetta (DE3) pLysS competent cells Novagen (Merck KGaA, Darmstadt, Germany) transformed with pQE81L plasmid carrying the human NS gene with a N-terminus 6-His tag were used to express and purify NS as reported in [15]. Briefly, bacterial cells were grown in Superior Broth (Molecular Dimension, Sheffield, UK) containing 100-µg/mL ampicillin and protein expression was induced with 0.2-mM isopropyl-β-D-thiogalactopyranoside at 17 °C for 16 h. NS was purified from the soluble fraction. Buffer A supplemented with cOmplete protease inhibitor cocktail (Hoffmann-La Roche, Basel, Switzerland) was added and the cell pellet was immediately sonicated. Crude extract was clarified by centrifugation and filtered before being loaded onto a 1 mL HiTrap Chelating HP column (GE Healthcare, Chicago, IL, USA) previously equilibrated with buffer A. Protein was eluted in 100% buffer B. Monomers were further isolated by gel filtration using a Hi Load 16/60 Superdex200 column (GE Healthcare, Chicago, IL, USA) equilibrated in 10-mM Tris-HCl, 50-mM KCl, pH 8.0. All purification steps were performed at 4 °C and all chemicals were purchased from Sigma-Aldrich (St. Louise, MO, USA), unless differently specified. NS concentration was measured by optical absorption using an extinction coefficient at 280 nm of 37,360 cm^−1^ M ^−1^.

### 2.2. Compounds Mix Preparation

EMB powder was directly dissolved in 10-mM Tris HCl, 50-mM KCl, pH 8.0 until reaching saturation conditions and the solution was immediately centrifuged at 4 °C for 10 min at 20,000× *g* in order to remove the excess of insoluble EMB. Absorbance at 325 nm was measured and EMB concentration was determined using an extinction coefficient of 24,000 cm^−1^ M^−1^ at pH 7.4. Supernatant was then used to dilute NS.

EMB analogs were dissolved as described above and used at saturation condition, but it was not possible to determine the precise concentration by optical absorbance.

### 2.3. Polymerization Assays

Solution of 85-μM native NS dissolved in 10-mM Tris-HCl, 50-mM KCl, pH 8.0 in the presence or absence of compounds prepared as described in Section 2.2 were incubated at 45 °C. Aliquots were collected after 0, 1 and 3 h of incubation and analyzed by SEC and non-denaturing-PAGE.

### 2.4. COS-7 cells Culture and DNA Transfection

COS-7 cells were cultured and transfected as described before [17]. Briefly, cells were maintained in DMEM (D6546) supplemented with 5% *v/v* FBS and Glutamax (Life Technologies, Carlsbad, CA, USA) at 37 °C and 5% *v/v* CO_2_ in a humidified incubator. Transfections were performed in 6-well plates pretreated with 0.1-mg/mL poly L-lysine (Sigma-Aldrich, St. Louise, MO, USA). Typically, 4 µg plasmid DNA were introduced into each well mixed with 10 µL of Lipofectamine 2000 (Life Technologies, Carlsbad, CA, USA) in serum-free Opti-MEM I culture medium (Life Technologies, Carlsbad, CA, USA) following the manufacturer’s protocol. The culture medium was replaced with fresh medium 16 h after transfection and DMSO as a control or 10-µM EMB alone or plus 2.5-µM MG132 were added to the fresh medium. Cells were incubated for another 24 h before collection of cell lysates and culture media as described in the next paragraph.

### 2.5. SDS and Non-Denaturing PAGE and Western Blot Analysis of Cellular Samples

The cell pellet from each well of 6-well plates was lysed in 100 µL of Nonidet lysis buffer [150 mM NaCl, 50 mM Tris-Cl, pH 7.5, 1% *v/v* Nonidet P-40, plus protease inhibitor mixture (cOmplete, Hoffmann-La Roche, Basel, Switzerland)]. The soluble protein fraction was collected in the supernatant after centrifugation at 12,000× *g*, 4 °C for 15 min, and proteins in the insoluble pellet were extracted by heating at 95 °C in loading buffer (Tris-HCl 125 mM pH 6.8, 10% *v/v* glycerol, 0.02% *w/v* bromo-phenol blue, 5% *v/v* beta-mercaptoethanol and 2% *w/v* SDS). Forty micrograms of total protein from each lysate and the equivalent volume of each culture medium were mixed with the same loading buffer (without SDS and beta-mercaptoethanol for non-denaturing PAGE) and analyzed in 10% *w/v* acrylamide SDS-PAGE or 7.5% *w/v* acrylamide non-denaturing PAGE and western blot as described before [29,30]. The horseradish peroxidase signal was developed using the LiteAblot PLUS and TURBO extra sensitive chemiluminescent substrates (EuroClone, Pero, Italy) and exposed to film or visualized on a ChemiDoc system (BioRad, Segrate, Italy). Unless stated otherwise, reagents, buffers, culture media and serum for cell cultures were purchased from Sigma-Aldrich (St. Louise, MO, USA). Rabbit polyclonal anti-NS antibody was made in-house [7]; rabbit polyclonal anti-GAPDH antibody was from Abcam (Cambridge, UK).

### 2.6. Non-Denaturing Polyacrylamide gel Electrophoresis

Samples were prepared as previously described [31]. Briefly, aliquots of NS in the presence or absence of compounds were collected and mixed at a 1:1 volume ratio with non-denaturing loading buffer (250-mM Tris-HCl, 50% glycerol, 0.5% bromophenol blue, pH 6.8). Samples were separated into 7.5% non-denaturing polyacrylamide gels run at 90 V for 2 h at 4 °C. Gels were stained with Coomassie brilliant blue R-250.

### 2.7. Size exclusion Chromatography

Analytical size exclusion chromatography runs were performed using a Superdex200 10/300 GL column (GE Healthcare, Chicago, IL, USA) or Superdex200 Increase 10/300 GL column (GE Healthcare, Chicago, IL, USA) previously equilibrated in 10-mM Tris HCl, 50-mM KCl, pH 8.0 buffer.

### 2.8. Synthesis of EMB Analogs

All reagents and solvents were reagent grade or were purified by standard methods before use. Melting points were determined in open capillaries by a SMP3 apparatus and are uncorrected. ^1^H spectra were recorded on Bruker AMX 300 MHz (Bruker, Billerica, MA, USA) or Bruker AV600 (Bruker, Billerica, MA, USA) spectrometers at 298 K, using deuterated solvents commercially available. Chemical shifts were reported in parts per million (δ). The coupling constants (J) are reported in Hertz (Hz) and ^13^C NMR spectra were recorded on Bruker AMX 300 MHz (Bruker, Billerica, MA, USA) or Bruker AV600 (Bruker, Billerica, MA, USA) spectrometers.

Solvents were routinely distilled prior to use; anhydrous tetrahydrofuran (THF) and ether (Et_2_O) were obtained by distillation from sodium-benzophenone ketyl; dry methylene chloride was obtained by distillation from phosphorus pentoxide. All reactions requiring anhydrous conditions were performed under a positive nitrogen flow and all glassware was oven dried and/or flame dried.

Isolation and purification of the compounds were performed by flash column chromatography on silica gel 60 (230–400 mesh). Analytical thin-layer chromatography (TLC) was conducted on TLC plates (silica gel 60 F254, aluminum foil). Compounds on TLC plates were detected under UV light at 254 and 365 nm or were revealed using TLC stains as KMnO_4_, iodine or 10% phosphomolybdic acid (PMA) in ethanol.

Silica treated with oxalic acid (referred to as oxalic acid-precoated silica) was prepared as follows: standard silica was suspended overnight in a 0.1 M oxalic acid (aq) solution; then was filtered under vacuum, then dried in oven.

Compound **4** was obtained as reported in literature [32].

Compounds **8** and **11** were obtained as reported in literature [33].

Compounds **9** was obtained as reported in literature [34].

Compounds **2a**, **3a**, **10a** and **1a** (embelin) were prepared as reported in literature [35].

3-(bromo-propyloxi)-tert-butyl-dimethyl-silane was prepared as reported in literature [36].

#### 2.8.1. 3-allyl-2,5-dimethoxy-[1,4]benzoquinone (**2e**) and 3-allyl-2-hydroxy-5-methoxy-[1,4]benzoquinone (**3e**)

To a solution of compound **11** (82 mg, 0.36 mmol) in CH_3_CN (3.5 mL), a solution of ceric ammonic nitrate (CAN, 502 mg, 0.89 mmol) in CH_3_CN/H_2_O 7/3 (4 mL) was added dropwise at −10 °C. The reaction was stirred at room temperature for 2 h, diluted with Et_2_O (20 mL) and washed with water (8 mL) and brine. The organic layer was dried over anhydrous Na_2_SO_4_ and concentrated in vacuo. The crude was purified by flash column chromatography on oxalic acid-precoated silica gel using ETP: ethyl acetate 85:15 as eluent, to give compounds **2e** (53 mg, 71%) and **3e** (12 mg, 17%) as bright yellow solids. R_f_ = 0.64 (**2e**) and 0.22 (**3e**) in ETP: ethyl acetate 85:15).

**2e**: mp: 90 °C; ^1^H-NMR (300 MHz, CDCl_3_): δ 5.86–5.73 (2H, m), 5.11–5.04 (1H, dq, *J* = 17.12, 1.59 Hz), 5.03–4.98 (1H, dq, *J* = 10.0, 1.36 Hz), 4.07 (3H, s), 3.81 (3H, s), 3.21–3.18 (2H, dt, *J* = 6.48, 1.45 Hz). ^13^C-NMR (75 MHz, CDCl_3_): δ 183.87, 182.24, 159.18, 156.42, 134.51, 128.01, 116.67, 105.87, 61.79, 56.80, 27.55.

**3e**: mp: 100 °C; ^1^H-NMR (300 MHz, CDCl_3_): 5b: δ 7.28 (1H, s), 5.90–5.76 (2H, m), 5.15–5.08 (1H, dq, *J* = 17.23, 1.58 Hz), 5.04–5.00 (1H, dq, *J* = 9.95, 1.28 Hz), 3.86 (3H, s), 3.22–3.19 (2H, dt, *J* = 6.56, 1.14 Hz). (in agreement with [37]).

#### 2.8.2. 3-allyl-2,5-dihdroxy-1,4-benzoquinone (**1e**)

To a solution of **2e** (39 mg, 0.19 mmol) in EtOH (8.5 mL), 2-M NaOH (aq., 4.2 mL) was added. The reaction was heated at 70 °C for 2 h. The solution was cooled at room temperature, then was diluted with 2-M HCl (21 mL) and extracted with ethyl acetate (3 × 21 mL). The combined organic phases were washed with brine, dried over anhydrous Na_2_SO_4_ and concentrated in vacuo to give compound **1e** (34 mg) as a brown solid in quantitative yield. mp: 144–146 °C; ^1^H-NMR (300 MHz, DMSO-*d*_6_): δ 11.26 (2H, br s), 5.83–5.73 (2H, m), 5.03–4.94 (2H, m), 3.06 (1H, br s), 3.04 (1H, br s); ^13^C-NMR (75 MHz, DMSO-*d*_6_): δ 161.4 (× 2C), 135.00(× 2C), 115.8 (× 2C), 115.2, 104.51, 26.69.

#### 2.8.3. 3-butyl-2,5-dimethoxy-[1,4] benzoquinone (**2d**) and 3-butyll-2-hydroxy-5-methoxy-[1,4] benzoquinone (**3d**)

To a solution of **10d** (70 mg, 0.27 mmol, 1 eq) in CH_3_CN (2.75 mL) cooled to −10 °C, ceric ammonium nitrate (CAN, 383 mg, 0.68 mmol) in CH_3_CN: H_2_O 7:3 (2.7 mL) was added dropwise. After the addition, the solution was stirred at room temperature for 2 h. The solution was diluted with Et_2_O (15 mL) and washed with H_2_O and brine. The organic layer was dried over anhydrous Na_2_SO_4_ and the solvent was evaporated. The resulting crude material was purified by preparative-TLC chromatography in hexane: ethyl acetate 8:2 to give compound **2d** (20 mg, 35%) as an orange solid and compound **3d** (18 mg, 29%) as a dark brown oil. **2d**: mp: 94 °C. ^1^H-NMR (300 MHz, CD_3_OH-*d*_4_): δ 5.85 (1H, s), 3.87 (3H, s), 2.47 (2H, t, *J* = 7.2 Hz), 1.54–1.41 (2H, m), 1.40–1.25(2H, m), 0.93 (3H, t, *J* = 7.2 Hz). **3d**: ^1^H-NMR (300 MHz, CD_3_OH-*d*_4_): 5.74 (1H, s), 4.07 (3H, s), 3.82 (3H, s), 2.45 (2H, t, *J* = 7.2 Hz), 1.48–1.36 (2H, m), 1.35–1.22 (2H, m), 0.93 (3H, t, *J* = 7.2 Hz). **2d** and **3d** are intermediates for the synthesis of **1d** and were not tested against NS polymerization.

#### 2.8.4. 3-butyl-2,5-dihydroxy-1,4-benzoquinone (**1d**)

To a solution of **2d** (11 mg, 0.05 mmol) in EtOH (2.2 mL), 2-M NaOH (aq., 1.1 mL) was added. The reaction mixture was heated at 70 °C for 2 h.

The solution was cooled to room temperature, then was diluted with 2-M HCl (5.4 mL) and extracted with ethyl acetate (3 × 5 mL). The combined organic phases were washed with brine (10 mL), dried over anhydrous Na_2_SO_4_ and the solvent was evaporated to give compound **1d** (10 mg) as a red/brownish solid in quantitative yield. mp: 132–133 °C. ^1^H-NMR (300 MHz, CH_3_OH-*d*_4_): δ 11.20 (2H, bs), 5.78 (1H, s), 2.46 (2H,t, *J* = 7.4 Hz); 1.45–1.18 (4H, m), 0.92 (3H, t, *J* = 7.1 Hz).

#### 2.8.5. 3-heptyl-1,2,4,5-tetramethoxybenzene (**10b**)

To a solution of 1,2,4,5-tetramethoxybenzene **9** (130 mg, 0.66 mmol) in dry THF (6 mL), *n*-BuLi (1.6 M in hexane, 610 µL, 0.98 mmol) was added at room temperature. After 40 min. under stirring, 1-bromoheptane (154 µL, 0.98 mmol) was added dropwise, and the reaction mixture was stirred overnight. Then, a saturated solution of NH_4_Cl was added, and the aqueous phase was extracted with ethyl acetate. The combined organic phases were dried over Na_2_SO_4_. The solvent was removed under vacuum and the crude mixture was purified by flash column chromatography with hexane: ethyl acetate 90:10, to give 88 mg of compound **10b** in 45% yield. Colorless oil. ^1^H-NMR (300 MHz, CDCl_3_): δ 6.41 (s, 1H), 3.84 (s, 3H), 3.77 (s, 3H), 2.65–2.55 (m, 2H), 1.59–1.45 (m, 2H), 1.43–1.21 (m, 8H), 0.87 (t, *J* = 7.5 Hz, 3H); ^13^C-NMR (75 MHz, CDCl_3_): δ 148.9 (× 2C), 141.2 (× 2C), 131.1, 96.8, 60.9 (× 2C), 56.3 (× 2C), 31.8, 30.7, 29.9, 29.1, 24.6, 22.5, 14.1.

#### 2.8.6. 3-Heptyl-2,5-Dihydroxy-[1,4] Benzoquinone (**1b**)

To a solution of **10b** (70 mg, 0.24 mmol) in CH_3_CN (2.3 mL) a solution of CAN (323 mg, 0.59 mmol) in CH_3_CN: H_2_O 7:3 (2.5 mL) was added dropwise at −10 °C (salt-ice bath) over 10 min. The reaction was allowed to stir at rt for 3 h, concentrated in vacuo and diluted with ethyl acetate. The organic layer was washed with water, brine, dried over anhydrous Na_2_SO_4_. The solvent was removed under vacuum and the residue was used in the next reaction without further purification. The crude was dissolved in ethanol (8 mL) and 2-M NaOH (aq., 4mL) was added, then the reaction was heated to reflux for 4 h. Then, the mixture was concentrated under vacuum and was diluted with 2-M HCl (10 mL). The precipitate was filtered under vacuum and washed with hexane, to give compound **1b** (17 mg, 30% yield) as an orange solid. ^1^H-NMR (300 MHz, DMSO-*d_6_*): δ 11.04 (brs, 2H), 5.76 (s, 1H), 2.26 (t, *J* = 6.9 Hz, 2H), 1.40–1.29 (m, 2H), 1.28–1.13 (m, 8H), 0.83 (t, *J* = 7.2 Hz, 3H). ^13^C-NMR (75 MHz, DMSO-*d_6_*): 117.35, 103.8, 31.2, 28.9, 28.4, 27.6, 22.0, 22.0, 13.9.

#### 2.8.7. 3-nonyl-1,2,4,5-tetramethoxybenzene (**10c**)

To a solution of 1,2,4,5-tetramethoxybenzene **13** (160 mg, 0.81 mmol) in dry THF (7 mL), *n*-BuLi (1.6-M in hexane, 750 µL, 1.21 mmol) was added at room temperature. After 30 min. under stirring, 1-bromononane (231 µL, 1.21 mmol) was added dropwise, and the reaction mixture was stirred overnight. Then, a saturated solution of NH_4_Cl was added, and the aqueous phase was extracted with ethyl acetate. The combined organic phases were dried over anhydrous Na_2_SO_4_. The solvent was removed under vacuum and the crude mixture was purified by flash column chromatography with hexane: ethyl acetate 95:5, to give 121 mg of compound **10c** in 46% yield. Colorless oil. ^1^H-NMR (300 MHz, CDCl_3_): δ 6.41 (1H, s), 3.84 (6H, s), 3.76 (6H, s), 2.65–2.56 (2H, m), 1.60–1.44 (2H, m), 1.43–1.26 (12H, m), 0.87 (3H, t, *J* = 6.9 Hz). ^13^C-NMR (75 MHz, CDCl_3_): δ 148.8(× 2C), 141.1 (× 2C), 131.1, 96.8, 60.9 (× 2C), 56.2 (× 2C), 31.9, 30.7, 30.0, 29.5, 29.4, 29.3, 24.6, 22.5, 14.1.

#### 2.8.8. 3-nonyl-2,5-dihydroxy-[1,4] benzoquinone (**1c**)

To a solution of **10c** (82 mg, 0.25 mmol) in CH_3_CN (2.5 mL) a solution of CAN (347 mg, 0.63 mmol) in CH_3_CN: H_2_O 7:3 (2.7 mL) was added dropwise at −10 °C (salt-ice bath) over 15 min. The reaction was allowed to stir at rt for 3 h, concentrated in vacuo and diluted with ethyl acetate. The organic layer was washed with water, brine, dried over anhydrous Na_2_SO_4_. The solvent was removed under vacuum and the residue was used in the next reaction without additional purification. The crude was dissolved in ethanol (8 mL) and 2 M NaOH aq. (4mL) was added, then the reaction was heated to reflux for 4 h. Then mixture was concentrated under vacuum and was diluted with 2-M HCl (10 mL). The obtained precipitate was filtered under vacuum and washed with hexane, to give compound **1c** (27 mg, 40% yield) as a bright orange solid. ^1^H-NMR (300 MHz, DMSO-*d_6_*): δ 10.92 (brs, 2H), 5.66 (s, 1H), 2.25 (t, *J* = 7.7 Hz, 2H), 1.40–1.12 (m, 10H), 0.83 (t, *J* = 6.9 Hz, 3H). ^13^C-NMR (75 MHz, CDCl_3_): δ 117.0, 102.2, 31.8, 29.5, 29.5, 29.3, 27.9, 22.6, 22.5, 14.1.

#### 2.8.9. Tert-butyldimethyl-[3-(2,3,5,6-tetramethoxyphenyl)-propoxy]-silane (**10f**)

A solution of 1,2,4,5-tetramethoxybenzene **9** (750 mg, 3.78 mmol) in anhydrous THF (4.7 mL), under nitrogen atmosphere, was cooled to 0 °C, then *n*-BuLi 1.6-M (2.4 mL, 3.78 mmol) was added dropwise. The reaction was warmed to room temperature and stirred for 2 h. Then, the reaction was cooled to −80 °C and a solution of 3-(bromopropyloxy)-tert-butyldimethylsilane (1.44 g, 5.68 mmol) in anhydrous THF (4.7 mL) was added dropwise. The reaction was slowly warmed to room temperature and stirred for 22 h. NH_4_Cl sat. (20 mL) was added to the reaction and the solution was diluted and extracted with ethyl acetate. The combined organic phases were washed with H_2_O, brine and dried on anhydrous Na_2_SO_4_. The crude (1.767 g) was purified by flash column chromatography in hexane: ethyl acetate 9:1, to give compound **10f** (488 mg, 35% yield) as yellow-pale oil.

^1^H-NMR (300 MHz, CDCl_3_): δ 6.41 (1H, s), 3.84 (6H, s), 3.76 (6H, s), 3.69 (2H, t, *J* = 7.2 Hz), 2.67 (2H, m), 1.84–1.66 (2H, m), 0.90 (9H, s), 0.06 (6H, s). ^13^C-NMR (150 MHz, CDCl_3_): δ 148.8 (× 2C), 141.0 (× 2C), 130.6, 96.7, 63.4, 60.9 (× 2C), 56.2 (× 2C), 33.9, 25.9 (× 3C), 21.2, 18.3, -5.2 (× 2C).

#### 2.8.10. 2,5-dihydroxy-3-(3-hydroxy-propyl)-[1,4] benzoquinone (**1f**)

To a solution of **10f** (100 mg, 0.27 mmol, 1 eq) in CH_3_CN (2.7 mL) cooled to −10 °C, a solution of CAN (376 mg, 0.67 mmol, 2.5) in CH_3_CN: H_2_O 7:3 (1.9 mL) was added dropwise. The reaction was warmed to room temperature and stirred for 2 h., then it was diluted with H_2_O (10 mL) and extracted with ethyl acetate (2 × 15 mL). The combined organic phases were washed with H_2_O, brine and then dried over anhydrous Na_2_SO_4_. The crude was used in the next step without additional purification. The residue was diluted with CH_2_Cl_2_ (2.7 mL) and HClO_4_ (60% *v/v*, 4 drops) was added; the reaction was stirred at room temperature for 3 h. The mixture was diluted with CH_2_Cl_2_ (3 mL) and washed with water. The aqueous phase was further extracted with CH_2_Cl_2_ (3 × 4 mL). The combined organic phases were washed with brine, dried over anhydrous Na_2_SO_4_ and the solvent was evaporated under vacuum. The resulting crude was purified by preparative-TLC (CH_2_Cl_2_, 0.5% CH_3_OH + 0.5% CH_3_COOH) to give product **1f** (15 mg, 28% yield) as a brown solid, mp: 155 °C ^1^H-NMR (600 MHz, CDCl_3_): δ 5.94 (1H, s), 4.39–4.27 (2H, m), 2.53–2.40 (2H, m), 2.06–1.93 (2H, m). ^13^C-NMR (150 MHz, CDCl_3_): δ 182.1, 181.8, 155.9, 155.1, 114.5, 104.7, 68.2, 29.7, 29.3, 20.2, 17.3.

#### 2.8.11. (E)-methyl–4-(2,3,5,6-tetramethoxyphenyl)-but-2-enoate (**12**)

To a stirred solution of **11** (180 mg, 0.80 mmol) in dry acetone (14 mL), K_2_CO_3_ (115 mg, 0.83 mmol, 1.04 eq) and CH_3_I (57 µL, 0.91 mmol, 1.14 eq) were added under nitrogen atmosphere. The mixture was heated to reflux for 16 h. The solvent was evaporated, the crude was diluted with AcOEt (10 mL), washed with water (7 mL) and the aqueous layer was further extract with AcOEt (2 × 7 mL). The combined organic layers were washed with brine (15 mL), then dried over anhydrous Na_2_SO_4_ and concentrated in vacuo. The crude was purified by flash chromatography in hexane: ethyl acetate 9:1, to give 91 mg of 3-allyl-1,2,4,5-tetramethoxybenzene as brown oil (yield 48%, R_f_ = 0.36 in hexane: ethyl acetate 9:1.

To a stirred solution of 3-allyl-1,2,4,5-tetramethoxybenzene (50 mg, 0.21 mmol) in DCM (4.3 mL), methyl acrylate (57 µL, 0.63 mmol) and UMICORE M73 SIMes (8 mg, 0.01 mmol) were added under nitrogen atmosphere. The mixture was stirred 6 h at room temperature. The solvent was evaporated, and the resulting crude was purified by flash chromatography in hexane: ethyl acetate 9:1, to give 25 mg of **12** as a yellow oil. (yield 40%, R_f_ = 0.12 in hexane: ethyl acetate 9:1. ^1^H-NMR (300 MHz, CDCl_3_): δ 7.12 (1H, dt, *J* = 6.2 Hz, 15.5 Hz); 6.47 (1H, s), 5.75 (1H, dt, *J* = 1.8 Hz, 15.5 Hz), 3.85 (6H, s), 3.75 (6H, s), 3.67 (3H, s), 3.56 (2H, dd, *J* = 1.8 Hz, 6.2 Hz).

#### 2.8.12. (2E)-4-(2,5-dihydroxy-3,6-dioxocyclohexa-1,4-dienyl)-but-2-enoic acid (**5**)

To a solution of **12** (42 mg, 0.14 mmol) in CH_3_CN (1.4 mL) cooled to −10 °C, a solution of CAN (195 mg, 0.35 mmol) in CH_3_CN: H_2_O 7:3 (1.4 mL) was added dropwise. The reaction was warmed to room temperature and stirred for 3 h., then it was diluted with H_2_O (10 mL) and extracted with ethyl acetate (2 × 15 mL). The combined organic phases were washed with H_2_O, brine and then dried over anhydrous Na_2_SO_4_. The crude was used in the next step without further purification. The residue was diluted with CH_2_Cl_2_ (2.5 mL) and HClO_4_ (60% *v/v*, 4 drops) was added; the reaction was stirred at room temperature for 3 h. The mixture was diluted with CH_2_Cl_2_ (3 mL) and washed with water. The aqueous phase was further extracted with CH_2_Cl_2_ (3 × 4 mL). The combined organic phases were washed with brine, dried over anhydrous Na_2_SO_4_ and the solvent was evaporated under vacuum to give a mixture of (2*E*)-methyl 4-(2,5-dihydroxy-3,6-dioxocyclohexa-1,4-dienyl)-but-2-enoate (20 mg) as a brown solid used in the next step without further purification. ^1^H-NMR (300 MHz, acetone-*d_6_*): δ 6.93 (1H, dt, *J* = 6.6 Hz, 15.6 Hz); 5.95 (1H, s); 5.88 (1H, dt, *J* = 1.5 Hz, 15.6 Hz); 3.66 (3H, s); 3.31–3.37 (2H, m).

To a solution of the above quinone (15 mg, 0.06 mmol) in aq. 50% THF (2.6 mL), LiOH H_2_O (13 mg, 0.32 mmol) was added and the mixture was stirred overnight at rt protected from light. The solvent was evaporated, and the crude was diluted with 2-N HCl (10 mL) then extracted with ethyl acetate and washed with brine. The organic phases were dried over anhydrous Na_2_SO_4_, the solvent was then removed in vacuo to give 9 mg (87%) of the product **5** as a brown solid.

^1^H-NMR (300 MHz, CH_3_OH-*d_4_*): δ 6.92 (1H, dt, *J* = 6.4 Hz, 15.4 Hz); 5.95 (1H, s); 5.80 (1H, dt, *J* = 1.5 Hz, 15.4 Hz); 3.33–3.37 (2H, m).

#### 2.8.13. 1,2,4,5-tetramethoxy-3-(pent-4-en-1-yl)benzene (**10g**)

1,2,4,5-tetramethoxybenzene **9** (0.77 g, 3.89 mmol) was dissolved in dry THF (20 mL) and HMPA (0.07 mL) was added. The solution was cooled down to −40 °C, then a 1.6-M solution of n-BuLi in hexane (3.8 mL, 6.08 mmol) was added dropwise. The reaction mixture was stirred for 40 min. while it was allowed to warm up to room temperature, then it was cooled down again to −40 °C and 5-bromo-1-pentene (1 mL, 8.44 mmol) was added dropwise. The reaction mixture was slowly warmed up to room temperature and stirred for 27 h, after which it was quenched with a saturated solution of NH4Cl (30 mL). The organic layer was extracted with ethyl acetate and the combined organic phases were washed with brine and dried over Na2SO4, then concentrated in vacuo. The crude product was purified by flash column chromatography using a hexane-ethyl acetate (85:15) solution, providing 498 mg (48% yield) of compound **10g** as a transparent oil. 1H-NMR (300 MHz, CDCl3): δ (ppm) = 6.41 (s, 1H); 5.84 (m, 1H); 5.01 (d, *J* = 14.7 Hz, 1H) 4.97 (d, *J* = 11.0 Hz, 1H); 3.84 (s, 6H); 3.76 (s, 6H); 2.63 (t, *J* = 7.7 Hz, 2H); 2.10 (dd, *J* = 14.3, 7.1 Hz, 2H); 1.63 (quint, *J* = 7.8 Hz, 2H). 13C-NMR (75 MHz, CDCl3): δ (ppm) = 148.84, 141.06, 138.89, 130.68, 114.28, 96.73, 60.94, 56.23, 33.97, 29.78, 24.15.

#### 2.8.14. 2,5-dihydroxy-3-(pent-4-en-1-yl)cyclohexa-2,5-diene-1,4-dione (**1g**)

A solution of compound **10g** (147 mg, 0.55 mmol) in acetonitrile (5.5 mL) was cooled down to −7 °C with an ice and salt bath, and a solution of 0.25-M CAN (760.3 mg, 1.38 mmol) in a CH_3_CN and H_2_O solution (7:3) was added dropwise. The reaction mixture was stirred at room temperature for 2 h. At the end of the reaction Et_2_O (25 mL) was added and the organic layer was washed with water and brine, then it was dried on Na_2_SO_4_ and concentrated in vacuo. The crude product of this first step was dissolved in ethanol (25 mL) and NaOH_aq_ 2 M (12.2 mL) was added. The reaction mixture was warmed up to 70 °C and stirred for 3 h. After reaction completion the solution was cooled down to 0 °C and it was acidified to pH 1 with HCl 37%. The reaction mixture was then extracted with ethyl acetate, the combined organic phases were dried on Na_2_SO_4_ and concentrated in vacuo. Product **1g** (80.7 mg, 71% yield) was obtained as a dark orange solid without any further purification. ^1^H-NMR (300 MHz, CDCl_3_): δ(ppm) = 6.00 (s, 1H); 5.77 (m, 1H); 4.99 (m, 2H); 2.46 (t, *J* = 7.8 Hz, 2H); 2.07 (m, 2H); 1.57 (quint, *J* = 7.6 Hz, 2H). ^13^C-NMR (100 MHz, *d^6^*-DMSO): *δ* (ppm) = 139.54, 118.18, 115.94, 104.97, 34.24, 27.94, 22.76 (detected signals).

#### 2.8.15. 3-allyl-2,5-bis-((2-(dimethylamino)ethyl)amino)-1,4-benzoquinone (**6**)

To a solution of benzoquinone **2e** (40 mg, 0.19 mmol) in CH_2_Cl_2_ (1.9 mL) at 0 °C, HClO_4_ (60% *v/v*, 1.5 mL) was added dropwise. The reaction was stirred 6 h at 0 °C, then was diluted with CH_2_Cl_2_ (10 mL) and washed with H_2_O and brine, dried over anhydrous Na_2_SO_4_ and evaporated. Compound **3e** (42 mg) was used in the next step without any further purification.

To a solution of **3e** (37 mg, 0.19 mmol) in EtOH (13 mL), NaHCO_3_ (0.82 g, 51 eq) and *N*,*N*-dimethylethylenediamine (0.25 mL, 1.95 mmol, 97% *w/w*) were added dropwise under nitrogen atmosphere. The reaction was stirred at room temperature for 48 h. The solvent was evaporated and the crude mixture was purified with preparative-TLC (CH_2_Cl_2_: CH_3_OH 9:1 + 1% H_2_O) providing compound **6** (17 mg, 35% yield) as a purple solid. mp: 64 °C. ^1^H-NMR (300 MHz, CDCl_3_): δ 7.27–7.18 (m, 1H), 7.07–6.95 (m, 1H), 6.02–5.88 (1H, m), 5.26 (s, 1H), 5.04 (dq, *J* = 1.9 Hz, 10.3 Hz, 1H), 4.98 (dq, *J* = 1.9 Hz, 17.0 Hz, 1H), 3.61 (q, *J* = 5.8 Hz, 2H), 3.31(dt, *J* = 2.0 Hz, 5 Hz, 2H), 3.15 (q, *J* = 5.8 Hz, 2H), 2.55 (t, *J* = 6.1 Hz, 2H), 2.51 (t, *J* = 6.1 Hz, 2H), 2.25 (s, 3H), 2.24 (s, 3H).

#### 2.8.16. (E)-1,8-bis(2,3,5,6-tetramethoxyphenyl)oct-4-ene (**13**)

To a solution of compound **10g** (106 mg, 0.40 mmol) in dry dichloromethane (4 mL) was added Hoveyda–Grubbs 2^nd^ generation catalyst (14.3 mg, 0.02 mmol). The reaction mixture was stirred at reflux for 4 h., then the solvent was evaporated in vacuo. The crude product was purified by flash column chromatography with a hexane and ethyl acetate (8:2) solution, providing 85 mg (85% yield) of product **13** as a transparent oil. ^1^H-NMR (300 MHz, CDCl_3_): δδ(ppm) = 6.40 (s, 2H); 5.45 (tt, *J* = 9.3, 5.2 Hz, 2H); 3.83 (s, 12H); 3.75 (s, 12H); 2.59 (m, 4H); 2.06 (m, 4H); 1.57 (brs, 4H).^13^C NMR (100 MHz, CDCl_3_): δδ(ppm) = 148.82, 141.23, 130.96, 130.30, 96.98, 60.92, 56.31, 32.95, 30.59, 24.25.

#### 2.8.17. 1,8-bis(2,3,5,6-tetramethoxyphenyl)octane (**14**)

Compound **13** (70.7 mg, 0.14 mmol) was dissolved in methanol (2.8 mL), then 10% _w_ Pd/C (118 mg) was added. The reaction mixture was placed in H_2_ atmosphere and stirred at room temperature for 24 h. After reaction completion, the catalyst was filtered on a double layer of Celite, paying attention to keep it wet, then the solvent was evaporated. The crude product was purified by flash column chromatography with a hexane and ethyl acetate (70:30) solution to afford 41.6 mg (59% yield) of product **14** as a crystalline transparent solid. ^1^H-NMR (300 MHz, CDCl_3_): δδ(ppm) = 6.39 (s, 2H); 3.83 (s, 12H); 3.75 (s, 12H); 2.58 (m, 4H); 1.51 (m, 4H); 1.38 (brs, 4H); 1.33 (brs, 4H). ^13^C NMR (100 MHz, CDCl_3_): δδ(ppm) = 148.91, 140.98, 128.54, 97.01, 60.42, 56.87, 31.74, 29.43, 29.02, 25.12.

#### 2.8.18. (E)-3,3’-(oct-4-ene-1,8-diyl)bis(2,5-dihydroxycyclohexa-2,5-diene-1,4-dione) (**7a**)

A solution of compound **13** (94 mg, 0.19 mmol) in acetonitrile (1.9 mL) was cooled down to −7 °C with an ice and salt bath and a solution of 0.25-M CAN (510.9 mg, 0.93 mmol) in a CH_3_CN/H_2_O (7:3) mixture was added drop-by-drop. The reaction mixture was stirred at room temperature for 4 h, after which it was quenched with Et_2_O (10 mL). The organic layer was washed with water and brine, then it was dried on a Na_2_SO_4_ and concentrated in vacuo. The crude product was employed in the next step without any further purification; it was dissolved in NaOH_aq_ 2 M (8.36 mL), then the reaction mixture was warmed up to 70 °C and stirred for 4 h. After reaction completion, the solution was cooled down to 0 °C and it was acidified to pH 1 with HCl 37%. The reaction mixture was extracted with ethyl acetate, the combined organic phases were dried on Na_2_SO_4_ and concentrated in vacuo. The resulting brown solid was triturated in hexane, providing 58 mg of product **7a** (79% yield) as a brown powder. ^1^H-NMR (300 MHz, CD_3_OD): δδ(ppm) = 6.44 (s, 2H); 5.42 (brs, 2H); 2.41–2.33 (m, 4H); 2.03 (brs, 4H); 1.61 (brs, 4H). ^13^C NMR (100 MHz, *d^6^*-DMSO): δδ(ppm) = 176.92, 170.57, 130.12, 123.99, 118.81, 32.27, 29.50, 29.02, 26.81 (detected signals).

#### 2.8.19. 3,3’-(octane-1,8-diyl)bis(2,5-dihydroxycyclohexa-2,5-diene-1,4-dione) (**7b**)

A solution of compound **14** (39 mg, 0.08 mmol) in acetonitrile (1 mL) was cooled down to −7 °C with an ice and salt bath and a solution of 0.25-M CAN (274 mg, 0.5 mmol) in a CH_3_CN/H_2_O (7:3) mixture was added drop-by-drop. The reaction mixture was stirred at room temperature for 7 h. The reaction was quenched with Et_2_O (5 mL) and the organic layer was washed with water and brine, then it was dried on Na_2_SO_4_ and concentrated in vacuo. The crude product was employed in the next step without any further purification; it was dissolved in ethanol (3.6 mL) and NaOH_aq_ 2-M (3.5 mL) was added. The reaction mixture was then warmed up to 70 °C and stirred for 4 h. After reaction completion the solution was cooled down to 0 °C, acidified to pH 1 with HCl 37% and extracted with ethyl acetate. The combined organic phases were dried on anhydrous Na_2_SO_4_ and concentrated in vacuo. The resulting brown solid was triturated in a solution of hexane and ethyl acetate (8:2), providing 27 mg of product **7b** (87% yield) as a brown solid. ^1^H-NMR (300 MHz, CDCl_3_): δδ(ppm) = 8.06 (brs, 4H) 5.99 (s, 2H); 2.35 (t, *J* = 7.4 Hz, 4H); 1.62 (m, 4H); 1.29– 1.24 (m, 8H). ^13^C NMR (100 MHz, *d^6^*-DMSO): δδ(ppm) = 119.74, 110.56, 34.40, 30.12, 29.89, 25.54 (detected signals).

## 3. Results

### 3.1. NS Polymerization at Different Concentrations of EMB

**Figure 1:**
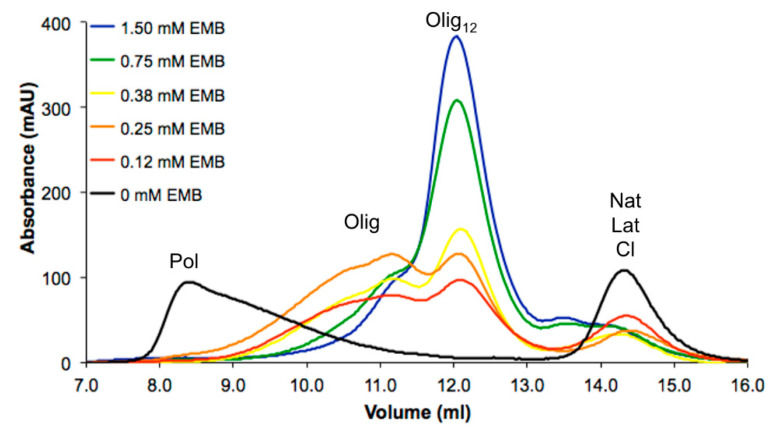
Effects of decreasing concentration of embelin (EMB) in heat-induced polymerization of neuroserpin (NS). Aliquots of 85-µM native NS were incubated 16 h at 45 °C in the absence (black) or presence of 1.5-mM (blue), 0.75-mM (green), 0.38-mM (yellow), 0.25-mM (orange) or 0.12-mM (red) EMB and analyzed by size-exclusion chromatography (SEC) using a Superdex 200 10/300 GL in 10-mM Tris HCl, 50-mM KCl, pH 8.0 buffer. Acronyms—Nat—native NS; Lat—latent NS; Cl—cleaved; Olig—oligomer; Pol—polymers.

In our previous study, embelin (EMB) was reported as the first molecule capable to interfere with the heat-induced in vitro polymerization of NS [25]. It was shown to destabilize Nat and Lat NS with a 1:1 EMB:NS binding ratio. All the experiments were performed in large excess of EMB (1.5 mM) compared to the concentration of NS (85 µM), but the effects of lower concentrations of EMB were not determined [25]. Here we test the effect of several dilutions of EMB on heat-induced in vitro polymerization of NS. A solution of 85-µM NS was incubated with EMB at concentrations ranging between 0 and 1.5 mM and NS polymerization was evaluated after 16 h of incubation at 45 °C by analytical size-exclusion chromatography (SEC, Figure 1)

In the absence of EMB, NS abundantly formed Pol which were eluted in the dead volume of the column (8.5 mL) as previously reported [25]; only a minor peak corresponding to monomeric Lat was eluted at 14.5 mL. In the presence of 1.5 mM EMB, the high-molecular weight Pol were not observed, but smaller oligomers eluted at 12.3 mL (Olig_12_), in keeping with the effect we previously reported in Saga et al. [25]. A very similar result was observed in the presence of 0.75-mM EMB, where the typical peak at 12.3 mL was predominant, but the peak eluted at 11.2 mL—corresponding to larger oligomers—was slightly more intense. The peak corresponding to Olig_12_ was present at all tested concentrations, but its intensity decreased proportionally to the amount of EMB added. The reduction of these species was paralleled by the appearance of larger aggregates as indicated by the appearance of two intense broad peaks eluting at 11.2 and 10.5 mL associated with larger oligomeric species (Olig).

### 3.2. EMB Promotes Proteasomal Degradation in Cell Lines Expressing NS

**Figure 2:**
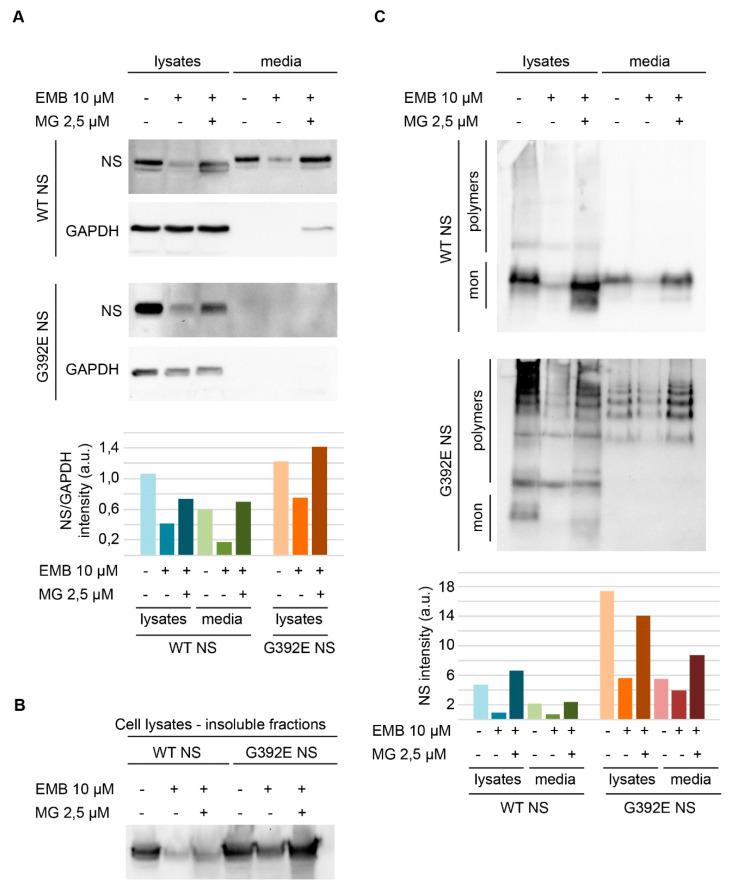
Embelin treatment results in a decreased level of wild type and polymerogenic G392E mutant NS that is reversed by proteasomal inhibition. (**A**) SDS-PAGE and western-blot analysis of lysates and culture media of cells transfected with wild type or G392E NS and treated with EMB (10 µM) alone or together with the proteasomal inhibitor MG132 (2.5 µM), as indicated in figure. GAPDH served as a loading control. Histogram shows the densitometric analysis of the western blot membranes, with NS intensity values normalized to GAPDH intensities for the corresponding lanes; (**B**) SDS-PAGE and western blot analysis of the insoluble proteins from post-nuclear pellets obtained from the same samples in A, extracted as described in the Methods section; (**C**) non-denaturing PAGE and western blot analysis of the same samples analyzed in A. The histogram shows the densitometric analysis of the western blot membranes; for the G392E NS samples, the value was calculated by addition of all the separate bands present in each lane.

The effect of EMB on polymerization was assessed in COS-7 cells transiently transfected for expression of wild type (WT) and G392E NS. Electrophoresis and western blot analysis of cells cultured for 24 h after transfection showed that, in the absence of EMB, WT NS was efficiently expressed and secreted (Figure 2A, WT NS panel, lanes 1 and 4). A small part of the protein was found in the insoluble fraction (Figure 2B, first lane), but most of it was contained in the soluble cellular fraction and culture medium as a native monomeric conformation, as revealed by non-denaturing PAGE (Figure 2C, WT NS panel, lanes 1 and 4). In keeping with previous studies [7], in cells expressing G392E NS most of the protein accumulated in the intracellular fraction, both in the soluble lysate (Figure 2A, G392E NS panel, first lane) and as insoluble aggregates (Figure 2B, lane 4); soluble intracellular and secreted G392E NS proteins were found mostly in the polymeric conformation (Figure 2C, G392E NS panel, lanes 1 and 4).

In parallel wells, transfected cells were treated with 10-µM EMB, collected after 24 h of incubation and analyzed similarly by SDS and non-denaturing electrophoresis and western blot. EMB promoted a reduction in NS signal for both WT and G392E NS transfected cells (Figure 2A,C, WT NS and G392E NS panels, lanes 2 and 5 and corresponding histograms). Unexpectedly, the polymerization pattern remained similar to the one observed in the absence of EMB, but with a weaker signal (Figure 2C, G392E NS panel, compare lane 1 to lane 2 and 4 to 5 and histogram). Inhibiting the proteasome in cells treated with 10-µM EMB by treating them at the same time with 2.5-µM MG132 caused a recovery in protein signals, which were restored to control levels (Figure 2, A and 2C, WT NS and G392E NS panels, compare lanes 3 and 6 with the other lanes and histograms). Moreover, the simultaneous treatment with EMB and MG132 exerted a modest toxicity in cells expressing NS, as shown by the presence of a low amount of the loading control protein GAPDH in the culture medium of cells expressing WT NS under the double treatment (Figure 2A, WT NS-GAPDH panel, lane 6). When COS-7 cells were transfected with a non-related neuronal protein, neuroligin 3, a reduction in protein levels after SDS-PAGE and western blot analysis of the cell lysates was also observed upon EMB treatment, with recovery to control levels when cells were treated simultaneously with EMB and MG132 as described for NS (results not shown).

### 3.3. Design and Synthesis of EMB Analogs

**Figure 3:**
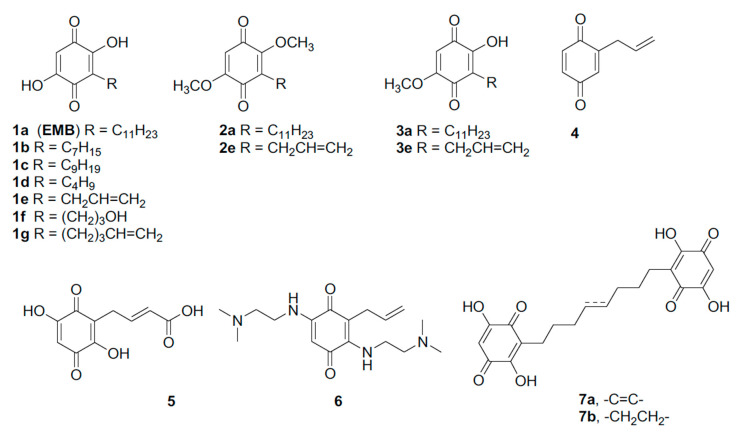
Structure of embelin (**1a**, EMB) and analogs (**1b**–**g**, **2a**,**e**, **3a**,**e**, **4**–**6**, **7a**,**b**).

In order to ameliorate EMB pharmacological properties and to explore the structure-activity relationship, EMB analogs were designed and synthetized (Figure 3). The low water solubility of EMB prevented crystallographic or NMR studies on the NS–EMB complex thus both chemical moieties of EMB, the lipophilic chain and the quinone ring, were systematically modified.

**Figure 4:**
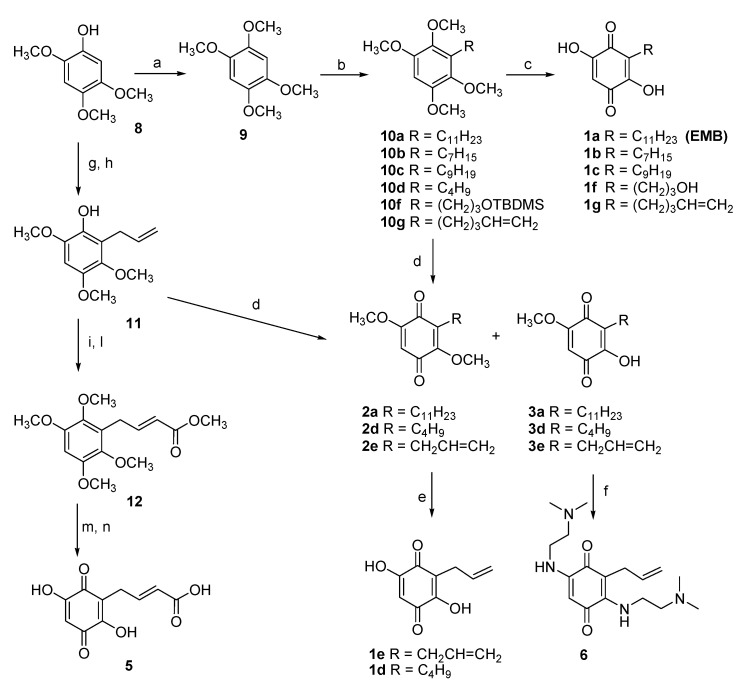
Synthesis of embelin (**1a**) and analogs **1b**–**1g**, **5** and **6**. Reagents and conditions: (a) CH_3_I, K_2_CO_3_, acetone, reflux, 86%; (b) for **10a**: *n*-BuLi 1.6 M, HMPA, THF, −40 °C, 1 h, then 1-bromoundecane, −10 °C, overnight, 28%; for **10b**: *n*-BuLi 1.6 M, THF, rt, 40 min. then heptylbromide, rt, overnight, 45%; for **10c**: *n*-BuLi 1.6 M, THF, rt, 30 min. then nonylbromide, rt, overnight, 46%; for **10d**: *n*-BuLi 1.6 M, HMPA, THF, −40 °C, 1 h, then 1-bromobutane, −10 °C, overnight, 31%; for **10f**: *n*-BuLi 1.6 M, THF, 0 °C to rt, 2 h, then 3-(bromopropyloxi)-*tert*-butyldimethylsilane −80 °C to rt, 22 h, 35%; for **10g**: *n*-BuLi 1.6 M, HMPA, THF, −40 °C to rt, 40 min., then 5-bromo-1-pentene, −40 °C to rt, 27 h, 48%; (c) i. CAN, CH_3_CN:H_2_O 7:3, −10 °C, then rt; ii. 2-M NaOH (aq.), EtOH, 70 °C, 2 h, yields over two steps for **1a**: 67%, **1b**: 30%, **1c**: 40%; **1g**: 71%; for **1f**: i. CAN, CH_3_CN: H_2_O 7:3, −10 °C, 2 h, ii. HClO_4_ 60%, CH_2_Cl_2_, rt, 3 h, 28% over two steps; (d) CAN, CH_3_CN: H_2_O 7:3, −10 °C then rt, yields **2a**: 36%, **3a**: 43%, **2e**: 71%, **3e**: 17%, **2d**: 35%, **3d**: 29%; (e) 2-M NaOH (aq.), EtOH, 70 °C, 2 h, **1e**: quant, **1d**: quant.; (f) NaHCO_3_, (CH_3_)_2_NCH_2_CH_2_NH_2_, EtOH, 48 h, rt **6**:35% (g) allyl bromide, K_2_CO_3_, acetone, 97%; (h) MW, 190 °C, 30 min, 92%; (i) CH_3_I, K_2_CO_3_, acetone, 16 h, reflux, 48%; (l) methyl acrylate, UMICORE M73 SIMes, CH_2_Cl_2_, 6 h, rt, 55%; (m) i. CAN, CH_3_CN: H_2_O 7:3, −10 °C, then rt; ii. HClO_4_ 70%, CH_2_Cl_2_, rt, 3 h, 61% over two steps; (n) LiOH·H_2_OH, THF: H_2_O 1:1, rt, overnight, 64%.

The role of the n-alkyl residue in position 3 was first, evaluated by modulation its length. Initially, shorter chains were inserted to reduce the lipophilicity with respect to the lead compound. The synthesis of embelin (**1a**) and its analogs **1b**–**1d** started from the common precursor 1,2,4,5-tetramethoxybenzene (**9**) [34] which was subjected to an ortho-metalation reaction in the presence of *n*-BuLi (Figure 4). To obtain the intermediates **10a**, **10d** and **10g** the ortho-metalation reactions were carried out in presence of hexamethylphosphoramide (HMPA) at −40 °C [38]. On the contrary, the intermediates **10c** and **10b** were obtained carrying out the reaction at room temperature and without HMPA [39].

The analog bearing an allyl chain at position 3 (compound **1e**) was prepared following a different strategy, based on the treatment of the phenol **8** with allyl bromide and subsequent microwave-assisted Claisen rearrangement to obtain the alkylated compound **11** [33].

Once obtained compounds **10a**–**d**, **10g** and **11,** the substituted benzene rings were oxidized to the corresponding quinones. EMB **1a** and quinones analogs **1b**, **1c** and **1g** were obtained by treatment with CAN, followed by hydrolysis of the crude intermediates with 2-M NaOH.

To evaluate the role of the free hydroxy groups on the EMB benzoquinone core, 2,5-dimethoxy and 2-hydroxy-5-methoxy-1,4-benzoquinones **2a** and **3a**, formed by CAN–mediated oxidation of **10a**, were isolated and purified. Following the same procedure, the analogs **2e** and **3e** were obtained. Treatment of 2,5-dimethoxy-1,4-benzoquinones intermediates **2e** with 2-M NaOH (aq.) in ethanol, gave 2,5-dihydroxy-1,4-benzoquinones **1e** in quantitative yield.

In order to increase the polarity and, hopefully, the water solubility with respect to the lead compound, a hydroxy group and a carboxylic group were introduced on the side chain, maintaining intact the 2,5-dihydroxy-1,4-benzoquinone scaffold.

With this purpose, (3-bromopropyloxy)-tertbutyldimethylsilane was used as electrophile after ortho lithiation substitution reaction, to obtain compound **10f** in 35% yield [40]. CAN-mediated oxidation and treatment with perchloric acid (60%) in dichloromethane of the resulting mixture of quinones gave the EMB analog **1f**.

To introduce a carboxylic group on the side chain, the allyl derivative **11** was methylated, then the resulting tetramethoxy intermediate was reacted with methyl acrylate in presence of the metathesis catalyst UMICORE M73 SIMes, to give compound **12** in 55% yield. CAN-mediated oxidation and treatment with perchloric acid (60%) in dichloromethane gave the corresponding quinone in 61% yield over two steps. Finally, hydrolysis of methyl ester with LiOH·H_2_O gave the acid **5**.

To explore the role of the 2,5-substituents on the quinone ring, the hydroxy groups were replaced by a *N*,*N*-dimethylethylenediamino residue. Compound **3e** was thus reacted with NaHCO_3_ and *N*,*N*-dimethylethyleneamine in ethanol for 48 h at room temperature [41], to give compound **6** in 35% yield.

Additionally, the quinone analog **4**, completely lacking substituents in position 2,5 on the ring, was prepared by CAN-mediate oxidation of 2-allyl-4-methoxyphenol [32].

**Figure 5:**
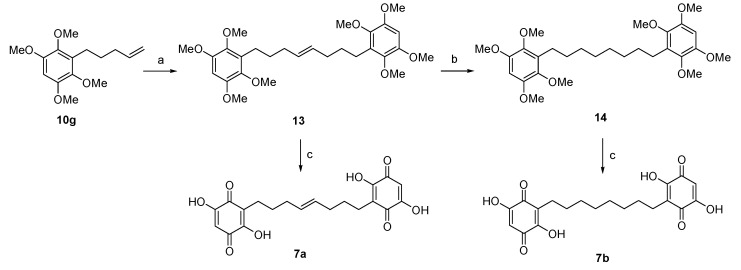
Synthesis of dimeric compounds **7a**,**b**. Reagents and conditions. (a) Hoveyda–Grubbs 2^nd^ generation catalyst, dry CH_2_Cl_2_, reflux, 5 h, 85%; (b) H_2_, Pd/C, MeOH, r.t., 24 h, 59%; (c) i. CAN, CH_3_CN: H_2_O 7:3, −10 °C to rt; 4 h ii. 2-M NaOH (aq.), EtOH, 70 °C, 4 h, yields over two steps for **7a**: 79%; for **7b**: 87%.

Finally, dimeric analogs of EMB were synthetized, aiming at an enhanced biologic activity with respect to the monomeric species and to increase the aqueous solubility due to the presence of two quinone rings in the final compounds (Figure 5). An olefin cross-metathesis reaction with Hoveyda–Grubbs second-generation catalyst was performed on compound **10g**, resulting in the dimer **13** with an 85% yield. The double bond of the dimeric compound was then reduced by hydrogenolysis to give compound **14** in 59% yield and both **13** and **14** were transformed via CAN-mediated oxidation to the corresponding bisquinones **7a** and **7b**, as outlined in Figure 5.

### 3.4. Effects of the EMB Analogs on NS Polymerization

**Figure 6:**
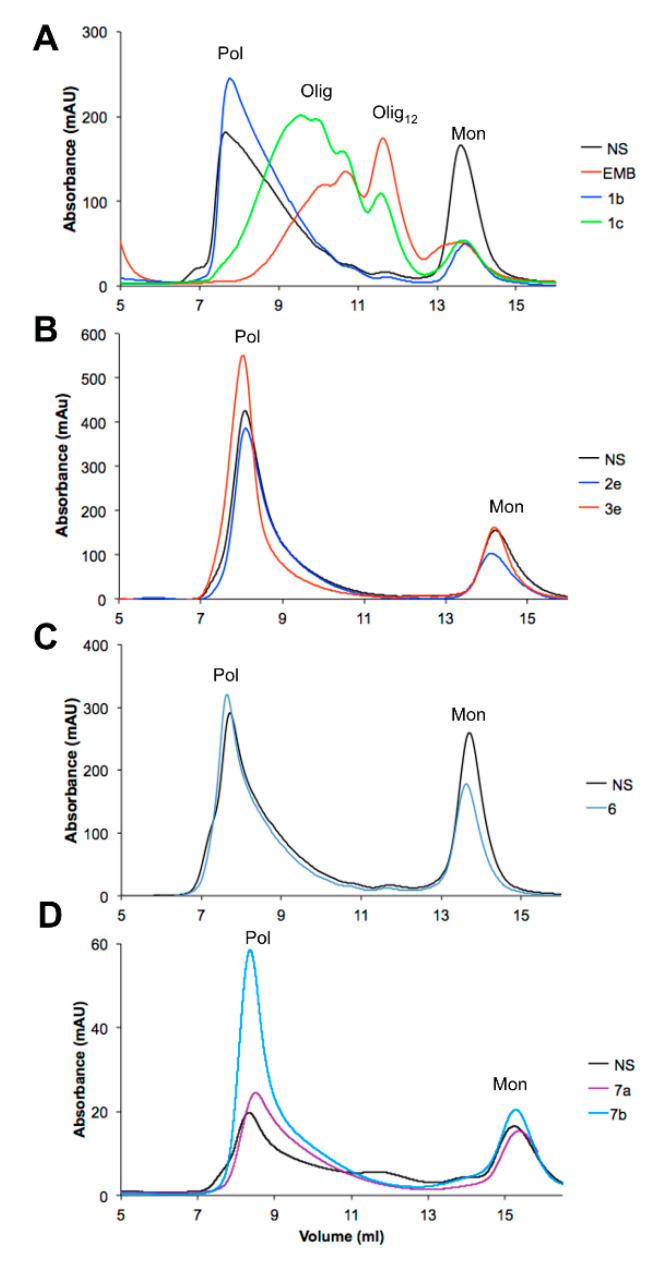
SEC analyses of EMB-analogs effect on heat-induced NS polymerization. Solutions of 85-µM NS was incubated 3 h at 45 °C in the presence or absence of EMB analogs at saturating concentration. The effect of representative compounds of (**A**) group 1, (**B**) group 2, (**C**) group 3, (**D**) group 3 is reported.

**Figure 7:**
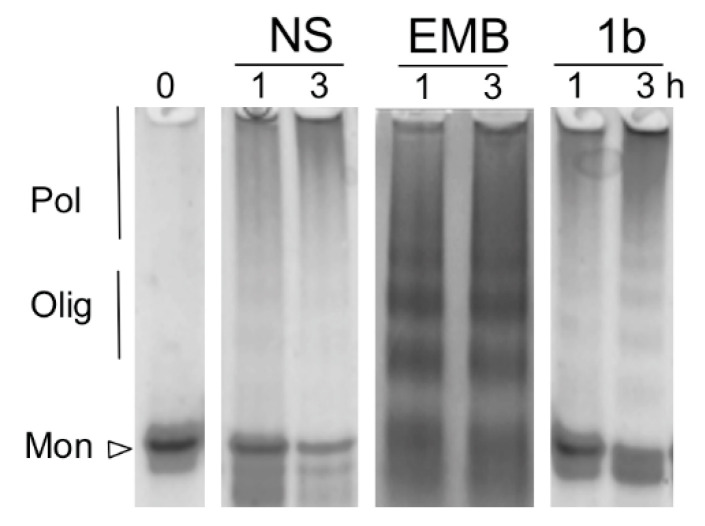
Non-denaturing PAGE analysis of EMB and 1b EMB analogs effect on heat-induced NS polymerization. Solutions of 85-µM NS were incubated for 3 h at 45 °C in presence or absence of EMB and analyzed by non-denaturing PAGE after 1 and 3 h of incubation.

All the EMB analogs synthetized were tested to assess their capability to interfere with heat-induced polymerization of NS. A solution of 85-µM NS was incubated at 45 °C in the presence or absence of each compound at saturating concentration. A solution of NS in the presence of EMB was used as an additional control. The effect of each compound after 3 h of incubation was assessed by SEC (Figure 6 and Table 1). After 1 or 3 h of incubation, an aliquot of the incubated mixture was taken and analyzed by non-denaturing PAGE (representative results are shown in Figure 7).

The first group of modifications was intended to modify the length of the alkyl tail in position 3. The effect of two representative EMB analogs (**1b** and **1c**) is reported in Figure 6A in comparison with unmodified EMB. As previously reported [25], EMB promoted the formation of Olig_12_ compared to the large polymers in its absence. Table 1 reports the distribution of different species in the presence or the absence of the compounds obtained by SEC analysis. In the presence of EMB almost no Pol were detectable, whereas 85% of the protein was in the oligomeric form (37% Olig and 48% Olig_12_). In the absence of any compounds 73% of NS was present as Pol that eluted in the dead volume. The same incubated mixtures of NS alone or NS in the presence of EMB were analyzed by non-denaturing PAGE (Figure 7), which clearly highlighted the differences between the two samples: in the first case the high-molecular weight Pol poorly entered the gel, whereas in the presence of EMB it was possible to separate different oligomeric species. A similar effect was observed for compound **1c**, which promoted the formation of oligomers, even though they eluted at higher column volume compared to the species formed in the presence of EMB. EMB analogs **1b**, **1d**, **1e**, **1f**, **1g** and **5** instead did not exert any significant effects on NS polymerization as reported in Table 1. Indeed, in the presence of these EMB analogs Olig_12_ were not present, whereas most of the protein formed Pol. **1c** on the contrary was still capable to prevent the formation of Pol but was less efficient compared to EMB to promote the formation of Olig_12_. Figure 7 shows a representative gel of coincubation of NS with EMB-derivative **1b**. According to SEC analysis, this compound promoted the formation of Pol as most represented species.

Next the role of the free hydroxyl group in positions C2 and C5 was evaluated. To this aim, two compounds were synthesized with the substitution of hydroxyl group in position 5 (**3a** and **3e**) or both hydroxyl groups in position 2 and 5 (**2a** and **2e**). Moreover, in compounds **2e** and **3e** also the alkyl tail was modified, and their effect is reported as representative of this group. For analogs **2e** and **3e**, no anti-polymeric action was detected as reported in Figure 6B. Table 1 clearly shows that in the presence of analogs **2e** and **3e** NS predominantly formed Pol (79% and 77%, respectively). Residual anti-polymeric activity was observed in the presence of **2a** and **3a** in which Olig and Olig_12_ were present, but still in a percentage lower than that in the presence of EMB. EMB derivative **3e** was slightly more effective than **3a** with 38% and 23% of protein present as Olig_12_, respectively (Table 1).

Compound **4**, completely lacking 2,5-hydroxy group on quinone scaffold with respect to compound **1e**, did not exert significant anti-polymeric activity, even though a slight amount of Olig_12_ was detected (11%, Table 1).

Moreover, also the replacement of both hydroxy groups at C2 and C5 with N,N-dimethylethylenediamino residues (**6**), did not produce any anti-polymerization effect (Table 1).

Finally, two different EMB dimers were tested (Figure 6D). Even in this case, the presence of the EMB analogs **7a** or **7b** did not exert significant effects on NS polymerization: as reported in Figure 6D and Table 1, traces of Olig were detected, but 56% and 70% of the protein were forming Pol, thus indicating that the polymerization pathway was not altered.

## 4. Discussion

The serpinopathies are a group of protein misfolding diseases characterized by the accumulation of serpin polymers in the endoplasmic reticulum of serpin-expressing cells [1]. To date no pharmacological treatment against the polymerization of any serpin is available, due to several reasons. Serpins are very plastic molecules which undergo major conformational changes both in physiologic and pathologic conditions [1]. In particular, the structure of serpin polymers is still under debate [2,20,21,22] preventing a structure-based drug design strategy. Moreover, the localization within the endoplasmic reticulum protects the serpin polymers from pharmacological molecules not able to penetrate the cellular membranes [2,3,5,6,7]. In the case of FENIB, a potential anti-polymerization molecule should also be able to pass the blood brain barrier. In this context, EMB displays several important properties: (I) it binds to all NS conformers with a stoichiometry of 1:1; (II) it is the first molecule capable to prevent NS Pol formation; (III) it can dissolve preformed polymers in vitro [25]; and (IV) it can cross the blood brain barrier [28].

In previous work [25], only the aqueous saturated solution of EMB was used to characterize its anti-polymerization properties, while here we describe for the first time the dose–response effects of EMB against NS polymerization. Our data indicate that while even the lowest concentration of EMB tested (0.12 mM) perturbs NS polymerization, lowering the concentration of EMB results in increasingly bulkier NS oligomers (Figure 1). When cell lines expressing a polymerogenic variant of NS (G392E NS, [4]) were treated with EMB, lower Pol levels were observed, but this effect was also apparent for WT NS and for a non-related protein (neuroligin 3, results not shown), and was reverted by simultaneous treatment with the proteasome inhibitor MG132, suggesting that the decrease in NS and neuroligin 3 levels was mainly due to the activation of the proteasome system or maybe to a general decrease in protein synthesis caused by EMB, rather than to a specific anti-polymerization activity. In fact, an increase in monomeric or oligomeric G392E NS in the presence of EMB was not observed after non-denaturing electrophoresis and western blot analysis, nor did we observe an increase in G392E NS secretion as expected for the monomeric protein. In particular, this compound was reported to regulates the apoptosis pathway through the inhibition of NF-kB. Even though a positive effect was observed, it was not directly associated with the remodulation of NS polymerization.

These observations prompted us to search for other small molecules with enhanced specificity. The low water solubility of EMB, its tendency to form micelles and its ability to cause NS oligomerization hampered crystallographic and NMR experiments; in turn, the lack of structural data on NS–EMB complex prevented a structural-based drug design strategy. Furthermore, NS is a very complex protein for in silico docking of small molecules: the Nat conformation is metastable and displays highly flexible regions and, moreover, it does not present any obvious deep cavity where small molecules could be accommodated. Thus, in order to identify other molecules with anti-polymerization properties, a systematic chemical modification of EMB was undertaken: the two main EMB moieties—the quinone ring and the lipophilic tail—were modified. None of the synthesized EMB analogs displayed an anti-polymerization effect distinctly better than the one reported for the lead compound. Interestingly, most of the modifications resulted in inactive compounds, indicating that the binding of EMB to NS is achieved by an already optimal interaction interface, and only a few minor chemical modification led to compounds that retained activity. All the chemical groups of EMB seem to be important for its interaction with NS, but the lipophilic tail seems to be crucial: the only tolerated modification is its shortening of two C atoms (**1c**), while all other modifications (shorter, more polar or longer tail, more rigid chain or dimeric structures) lead to inactive molecules.

The substitution of one or two hydroxyl groups on the quinone ring with *O*-methyl groups (compounds **2a** and **3a**, respectively) resulted in active compounds. In presence of compound **2a** NS forms smaller oligomers than the ones formed in presence of **3a**, but EMB remained better inhibitor compared with these two molecules. This observation indicates that compounds with hydroxyl groups in such positions have a higher anti-polymerization activity, thus in that respect EMB presents optimized substituent on the quinone ring. Moreover, the comparison of effects exerted by the couple **2a/3a** and **2e/3e** confirmed the importance of the alkyl tail in the inhibition process. Indeed, these couple of compounds differs only in the tail structure. In general, other modifications of the substituents on the quinone ring resulted in inactive molecules and, in particular, all analogs bulkier than EMB lost activity. Altogether, our results confirmed that, to date, EMB is the best inhibitor of NS polymerization in vitro.

EMB was reported to bind to PAI-1, PAI-2 and PN1, three members of the serpin family: EMB is an antagonist of the inhibitory abilities of these serpins against their target proteases, while no effects on polymerization were reported [42]. A study about EMB-derived molecules tested for PAI-1 identified the hydroxyl groups at C2 and C5 and the length of the alkyl chains at C3 as determinant for inhibitory potency and some EMB analogs displaying relevant inhibition properties were identified [43]. Still, in that study the effects of EMB and its analogs were tested with regards to modulation of serpin inhibitory activity against its target protease and polymerization was not addressed. In a different report [44], even very conservative modifications of the structure of EMB abolished its ability to interfere with α-glucosidase, a protein involved in diabetes mellitus, in analogy with what we observed in the present work.

In summary, in this work we show that the interaction between EMB and NS is already so specific that all the analogs produced were found to be either inactive or with a lower anti-polymerization activity than EMB itself. The activity of EMB decreased with decreasing concentration, however all tested EMB concentrations were able to reduce NS polymerization. Finally, the effect observed in a cell model was the reduction of NS protein level through a non-NS specific effect due to an increase in proteasomal degradation. Overall, the present data are encouraging; however, future strategies to better shuttle EMB into the endoplasmic reticulum of neurons to achieve high local concentrations will likely lead to stronger and more specific anti-polymerization effects.

## Figures and Tables

**Table 1:** EMB-analogs effect on NS heat-induced polymerization. The effect of EMB analogs on NS heat-induced polymerization at saturating concentration after 3 h of incubation at 45 °C was evaluated by SEC analysis and the amount of each species was calculated. Pol—NS polymers eluting in the dead column volume; Olig—oligomers eluting at 10–11 mL; Olig12—oligomers typically formed in presence of EMB eluting at 12.3 mL; Mon—monomeric NS eluted at 14–15 mL; Control—NS incubated in absence of potential inhibitors; All compounds were tested at saturating concentration.

	Pol	Olig	Olig12	Mon
**Control**	73%	0%	5%	22%
**EMB, 1a**	2%	37%	48%	14%
**1b**	80%	0%	3%	17%
**1c**	10%	50%	27%	13%
**1d**	54%	0%	0%	46%
**1e**	66%	0%	0%	34%
**1f**	69%	0%	0%	31%
**1g**	52%	0%	0%	48%
**2a**	3%	44%	23%	30%
**3a**	7%	35%	38%	20%
**2e**	79%	0%	0%	21%
**3e**	77%	0%	0%	23%
**4**	73%	0%	11%	16%
**5**	76%	0%	0%	24%
**6**	66%	0%	0%	34%
**7a**	56%	8%	0%	35%
**7b**	70%	06%	0%	24%
